# Attenuation of Splanchnic Autotransfusion Following Noninvasive Ultrasound Renal Denervation: A Novel Marker of Procedural Success

**DOI:** 10.1161/JAHA.118.009151

**Published:** 2018-06-12

**Authors:** Manish Saxena, Tariq Shour, Mussadiq Shah, Christopher B. Wolff, Peter O. O. Julu, Vikas Kapil, David J. Collier, Fu Liang Ng, Ajay Gupta, Armida Balawon, Jane Pheby, Anne Zak, Gurvinder Rull, Benjamin O'Brien, Roland E. Schmieder, Melvin D. Lobo

**Affiliations:** ^1^ Barts BP Centre of Excellence Barts Heart Centre St Bartholomew's Hospital London United Kingdom; ^2^ William Harvey Research Institute Centre for Clinical Pharmacology Barts NIHR Cardiovascular Biomedical Research Unit Queen Mary University London London United Kingdom; ^3^ Department of Nephrology and Hypertension University Hospital University of Erlangen/Nuremberg Erlangen Germany

**Keywords:** autonomic function, blood pressure, hypertension, renal denervation, sympathetic nervous system, ultrasound, Valsalva maneuver, High Blood Pressure, Hypertension, Autonomic Nervous System, Clinical Studies, Ultrasound

## Abstract

**Background:**

Renal denervation has no validated marker of procedural success. We hypothesized that successful renal denervation would reduce renal sympathetic nerve signaling demonstrated by attenuation of α‐1‐adrenoceptor‐mediated autotransfusion during the Valsalva maneuver.

**Methods and Results:**

In this substudy of the Wave IV Study: Phase II Randomized Sham Controlled Study of Renal Denervation for Subjects With Uncontrolled Hypertension, we enrolled 23 subjects with resistant hypertension. They were randomized either to bilateral renal denervation using therapeutic levels of ultrasound energy (n=12) or sham application of diagnostic ultrasound (n=11). Within‐group changes in autonomic parameters, office and ambulatory blood pressure were compared between baseline and 6 months in a double‐blind manner. There was significant office blood pressure reduction in both treatment (16.1±27.3 mm Hg, *P*<0.05) and sham groups (27.9±15.0 mm Hg, *P*<0.01) because of which the study was discontinued prematurely. However, during the late phase II (Iii) of Valsalva maneuver, renal denervation resulted in substantial and significant reduction in mean arterial pressure (21.8±25.2 mm Hg, *P*<0.05) with no significant changes in the sham group. Moreover, there were significant reductions in heart rate in the actively treated group at rest (6.0±11.5 beats per minute, *P*<0.05) and during postural changes (supine 7.2±8.4 beats per minute, *P*<0.05, sit up 12.7±16.7 beats per minute, *P*<0.05), which were not observed in the sham group.

**Conclusions:**

Blood pressure reduction per se is not necessarily a marker of successful renal nerve ablation. Reduction in splanchnic autotransfusion following renal denervation has not been previously demonstrated and denotes attenuation of (renal) sympathetic efferent activity and could serve as a marker of procedural success.

**Clinical Trial Registration:**

URL: https://www.clinicaltrials.gov. Unique identifier: NCT02029885.

**Clinical Perspective**

**What Is New?**

- We describe a novel strategy to evaluate the procedural success of an ultrasound‐based renal denervation platform by use of noninvasive autonomic function testing (with real‐time beat‐to‐beat blood pressure monitoring) to delineate changes in heart rate, mean arterial pressure, and autotransfusion responses to the Valsalva maneuver.
- This methodology, which is easily reproducible, offers an attractive alternative to the current approaches to assess sympathetic nervous system activity such as microneurography and norepinephrine spillover techniques.

**What Are the Clinical Implications?**

- We demonstrate that renal denervation resulted in significant reductions in both heart rate and autotransfusion response to the Valsalva maneuver, suggesting that external ultrasound renal denervation is effective, and that greater blood pressure reduction alone may be misleading as a sole marker of response to an interventional procedure compared with sham.
- This study makes a significant contribution to the debate over the utility of the sham procedure in device therapy of hypertension trials and emphasizes the need to find noninvasive markers of procedural success of renal denervation.

The recent past has seen the emergence of a variety of promising novel nonpharmacological device‐based therapies for treating hypertension.^1^ Most of these new technologies target sympatho‐modulation either through renal nerve ablation (renal denervation or RDN) or through electrical carotid sinus stimulation (baroreflex activation therapy), although other technologies may target mechanical aspects of the circulation through the creation of a central iliac arteriovenous anastomosis.^2^, ^3^, ^4^, ^5^, ^6^, ^7^ At present, RDN has been studied the most and is thought to lower blood pressure (BP) by reducing central sympathetic drive and in turn, reducing sympathetic outflow to kidneys.^8^, ^9^

A major concern with RDN is that there is no procedural marker of success and it is still regarded as a “black box” procedure whereby the operator is unaware at the time of intervention whether or not the renal nerves have been successfully ablated.^10^ Recent data identifying more precisely the location of human renal sympathetic nerves have better informed the procedure to improve delivery of ablation foci in the most appropriate territory, but this still does not address whether or not the technology has achieved its intended aim of renal nerve destruction.^11^

The use of physiological measures and biomarkers to confirm procedural success of renal nerve ablation, such as muscle sympathetic nerve activity and renal norepinephrine spillover, has been proposed but remains contentious.^12^, ^13^, ^14^ Renal nerve stimulation‐induced BP increase pre‐ and post‐RDN has been shown to be a good marker to predict BP response and assess efficacy of RDN but requires further validation.^15^ Thus, there remains a pressing need to identify valid biomarkers to confirm procedural success that are noninvasive and easy to use in everyday clinical practice.

The objectives of this substudy were to investigate changes in physiological and autonomic nervous system parameters with noninvasive testing in subjects with resistant hypertension randomized to having bilateral renal nerve ablation using externally delivered focused ultrasound or sham therapy. Specifically, our aim was to test the hypothesis that successful renal nerve ablation would result in attenuation of renal sympathetic nervous system (SNS) activity measured as a lowering of global sympathetic tone and also as a reduction in sympathetically mediated autotransfusion during (late) phase II of the Valsalva maneuver, which depends in part upon renal capsular contraction mediated through renal SNS signaling.^16^

## Methods

The data, analytic methods, and study materials will not be made available to other researchers for purposes of reproducing the results or replicating the procedure.

### Study Design and Subjects

This study is a single‐center substudy of the Wave IV Study: Phase II Randomized Sham Controlled Study of Renal Denervation for Subjects With Uncontrolled Hypertension study, an international, multicenter, randomized, sham‐controlled, double‐blind clinical trial of RDN in subjects with treatment‐resistant hypertension.^17^ The study had approval from a Central Research Ethics Committee and all participants gave written informed consent to take part in the main study and the substudy. Subjects between 18 and 80 years of age with uncontrolled primary arterial hypertension, defined as office SBP ≥160 mm Hg while taking 3 or more antihypertensive medications at the maximally tolerated doses, one of which was a diuretic, were enrolled in the study. Other inclusion criteria were a 24‐hour ambulatory BP monitoring average SBP of ≥135 mm Hg and no medication changes within the month before baseline. Subjects were excluded from the study if they had a secondary cause of hypertension; had clinically significant renal artery stenosis; had previously undergone RDN; had known severe pulmonary hypertension; had experienced a myocardial infarction, unstable angina, or cerebrovascular event within the 6 months before baseline; had hemodynamically significant valvular heart disease; had a body mass index (BMI) >35 kg/m^2^; or were pregnant, nursing, or intended to become pregnant during the study period. Furthermore, factors that may preclude clear visualization of the renal parenchyma and renal artery were also exclusion criteria.

Patients were centrally randomized in a 1:1 ratio to receive either the active RDN treatment or the sham control, both administered using the Surround Sound System. The treatment consisted of bilateral RDN using therapeutic levels of ultrasound energy. The sham consisted of bilateral sham treatment using diagnostic levels of ultrasound energy. A detailed description of the study design and interventional/sham procedures can be found in Data S1.

### Office BP

The office BP measurements were recorded with an automated validated Omron M6 Comfort Intellisense device (Omron Corp, Tokyo, Japan) in triplicate, taken with subjects in a seated position, after 10 minutes of rest. The 3 BP measurements were taken 1 minute apart and the average of the 3 measurements was calculated.

At the screening visit, BP was measured in triplicate in both arms and the arm with the highest BP was used for all subsequent readings for the entire duration of the study. At any visit, if the variability in the 3 BP readings was >15 mm Hg, another set of 3 readings was taken.

### Ambulatory BP

Ambulatory BP was measured using oscillometric Space Labs 90207‐1Q monitor (Spacelabs Healthcare, Hertford, UK). BP was recorded every 30 minutes during the daytime and every 60 minutes during the night. Technical success of the ambulatory BP monitoring required 70% of BP recordings to be valid.

### Autonomic Function Tests

Autonomic function tests were conducted in a standardized controlled environment with a room temperature of 23±1°C, diffused lighting, and no background noise. The procedure was discussed in detail with the subjects beforehand so that they were well familiar with the autonomic study protocol. They were relaxed and comfortable during the procedure.

The ECG used to derive cardiovascular indices was recorded via 3 chest electrodes conforming to Einthoven's Lead II configuration. Noninvasive arterial BP waveform was recorded continuously using volume‐clamp photoplethysmography through a finger cuff, which is part of the Finapres™ system (Finapres Medical Systems, Amsterdam, The Netherlands).^18^ All raw data including BP waveforms were used to derive real‐time autonomic indices by a NeuroScope using the VaguSoft™ software (Medifit Instruments Ltd, London, UK). The instantaneous heart rate (HR, in beats per minute [bpm]) was calculated continuously in real‐time from the intervals between consecutive electrocardiographic R‐waves (R‐R intervals) to facilitate measurement of sudden or rapid changes in HR.

### Postural Studies

Baseline autonomic status was recorded with subjects in a reclined position (patient at 45 degrees) and subsequently supine position for at least 3 minutes with the mean BP varying by <5 mm Hg.

Subjects then sat upright from the supine position with neck and legs held in a straight position while looking directly ahead, for a duration of 3 minutes. This invokes the isometric contraction of neck and trunk muscles. At the end of sit‐up, subjects returned to a reclined and supported position.

### Valsalva Maneuver

Subjects sat on a chair, leaning forward onto an examination couch with both hands rested. This posture is designed to exert mild pressure on the femoral and iliac veins and restrict the venous return from the lower limbs. The Valsalva test assembly consists of an air pressure gauge and detachable paper mouthpiece (a vitalograph tube). The mouthpiece has an aperture at the bottom of the tube to permit a small amount of air to escape, allowing constant pressure to be maintained.

Subjects blew into the tube assembly by either placing the lips tightly around the mouthpiece or squeezing the lips inside the mouthpiece. Both methods sealed‐off air leakage while blowing into the mouthpiece.

After successful practices, subjects were observed at rest to ensure that mean arterial BP (MAP) was varying by <5 mm Hg to make sure that subjects were relaxed and had recovered from the previous practice maneuver. To start the maneuver, subjects took a deep breath and then blew into the mouthpiece without air leaks to bring the pointer aligned with the black line in middle of the slave meter (denoting 40 mm Hg) for 15 s. At the end of the 15 s, the subjects stopped blowing into the mouthpiece, and resumed normal breathing at their normal tidal volume.

This maneuver was repeated to obtain 3 clear recordings. The Stable segment of MAP for 20 R‐R intervals just preceding the Valsalva maneuver was compared with the 4 phases and 5 peaks of the Valsalva maneuver. The best of 3 well‐performed maneuvers with all 4 phases of BP changes was chosen for analysis.

### Follow‐Up

Subjects were assessed at baseline and 6 months post‐treatment. At these visits, office BP measurements were recorded as described above. During the 6‐month visit, a 24‐hour ambulatory BP monitoring was repeated, together with the same battery of autonomic function tests described above. At this time point, both subjects and the study team were still blinded to the intervention allocated to individual subjects.

### Statistical Analysis

To determine significant differences between groups, 2‐tailed independent Student *t* tests were used. Data are represented as mean±SD, unless otherwise indicated. When looking at within‐group differences between time points, 2‐tailed dependent Student *t* tests were used. A significant difference was defined as having a *P*<0.05.

All authors had access to the study data and take full responsibility for its integrity and data analysis.

## Results

### Demographics

The demographics of the study cohort are summarized in Table 1. Out of the 23 subjects analyzed, 2 were excluded from the autonomic function analysis. One patient was lost to follow‐up and another did not undergo autonomic function testing before treatment. There were no statistical differences between the groups with regard to sex, age, body mass index, smoking status, comorbidities of type 2 diabetes mellitus and cardiovascular disease, and antihypertensive medications prescribed.

**Table 1:** Demographics

	Treatment	Sham	Total
N	12	11	23
Male	9	6	15
Female	3	5	8
Average age (y)	57.2±10.3	61.9±10.6	59.5±10.5
Average BMI	31.7±3.4	29.7±4.8	30.7±4.2
Smoking history	6	4	10
Type 2 DM	1	3	4
CVD	4	1	5
Average BP medications	4.7±1.5	4.7±1.2	4.7±1.4
ACEI/ARB	11	9	20
CCB	10	8	18
Diuretics	12	11	23
β‐Blockers	4	6	10
α‐Blockers	8	7	15
MR antagonist	6	5	11

Data are expressed as mean±SD. ACEI indicates angiotensin‐converting enzyme inhibitor; ARB, angiotensin receptor blocker; BMI, body mass index; BP, blood pressure; CCB, calcium channel blocker; CVD, cardiovascular disease; DM, diabetes mellitus; MR, mineralocorticoid receptor.

### Office Blood Pressure

The office BP data are summarized in Table 2. There was a significant reduction in office SBP in both the treatment (16.1±27.3 mm Hg, *P*<0.05) and sham groups (27.9±15.0 mm Hg, *P*<0.001) and a significant reduction in office diastolic BP in only the sham group (15.4±14.9 mm Hg, *P*<0.05), but no statistical significance between the groups.

**Table 2:** Changes in Office Blood Pressure and Ambulatory Blood Pressure Between Baseline and 6 Months Postprocedure

	Treatment (n=12)			Sham (n=11)		
	Baseline	6 Mo	*P* Value	Baseline	6 Mo	*P* Value
Office						
SBP	170.7±11.2	154.7±26.9	0.04	180.4±14.3	152.4±25.7	0.001
DBP	99.9±14.0	91.4±14.8	0.06	101.0±16.8	85.7±14.5	0.02
HR	78.5±13.0	72.5±11.6	0.03	76.1±15.8	75.2±17.1	0.94
Ambulatory						
Day SBP	157.7±9.5	147.1±17.9	0.06	162.8±5.6	152.0±13.5	0.01
Day DBP	93.6±11.9	85.5±14.0	0.06	90.9±14.5	84.5±10.9	0.03
Day MAP	116.3±8.2	107.6±13.8	0.08	116.0±8.9	108.2±9.0	0.02
Day HR	76.4±8.1	72.1±6.0	0.05	75.4±13.9	75.9±15.0	0.80
Night SBP	146.8±14.2	141.8±19.7	0.30	153.8±12.4	141.9±20.2	0.04
Night DBP	82.8±11.0	81.0±14.7	0.61	83.8±12.3	76.5±12.3	0.05
Night MAP	105.9±10.6	102.6±14.8	0.40	109.0±8.9	99.6±12.5	0.02
Night HR	69.9±7.8	68±7.9	0.44	70.4±10.2	71.2±13.5	0.73

Data are expressed as mean±SD. *P* values compares the 2 time points. DBP indicates diastolic blood pressure; HR, heart rate; MAP, mean arterial pressure; SBP, systolic blood pressure.

### Ambulatory Blood Pressure

In the treatment group, reductions in daytime SBP, diastolic BP, and MAP were numerically similar to the sham group but did not achieve statistical significance. In the sham limb, there was a significant reduction in daytime SBP (10.8±12.0 mm Hg, *P*<0.05), daytime diastolic BP (6.5±8.6 mm Hg, *P*<0.05), daytime mean arterial pressure (MAP) (7.8±9.7 mm Hg, *P*<0.05), night‐time SBP (11.9±16.3 mm Hg, *P*<0.05), and night‐time MAP (9.4±11.3 mm Hg, *P*<0.05). There were no statistical differences between the treatment and sham groups. The 24‐hour ambulatory BP data are summarized in Table 2.

### Heart Rate

There was a significant reduction in resting HR in only the treatment group (6.0±11.5 bpm, *P*<0.05) compared with baseline. In the treatment limb, significant reduction was observed in the daytime HR (4.3±6.6 bpm *P*<0.05) during 24‐hour BP monitoring with no significant HR changes in the sham group during day or night. The office and 24‐hour HR data are summarized in Table 2.

### BP and HR responses to Autonomic Function Tests

The autonomic function test results are summarized in Table 3. During postural studies, there was a significant reduction in HR in the treatment group in the reclined position (6.9±9.1 bpm, *P*<0.05) and supine position (7.2±8.4 bpm, *P*<0.05) following RDN (Table 3). There was no statistical difference between the treatment and sham groups in the change over time. There was also a significant reduction in both HR (12.7±16.7 bpm, *P*<0.05) and MAP during the sit up (10.6±13.7 mm Hg, *P*<0.05) at 6 months follow‐up compared with baseline. No statistically significant changes were identified in the sham group.

**Table 3:** Changes With Autonomic Function Tests Between Baseline and 6 Months Postprocedure

	Treatment (n=10)			Sham (n=11)		
	Baseline	6 Mo	*P* Value	Baseline	6 Mo	*P* Value
Postural studies						
SBP reclined	153.5±22.6	137.0±29.1	0.18	158.0±18.9	152.0±38.1	0.50
DBP reclined	70.8±20.9	62.2±19.4	0.14	65.0±18.7	63.8±21.1	0.77
MAP reclined	98.3±19.9	87.1±21.5	0.15	96.0±14.0	93.2±20.8	0.58
HR reclined	79.6±14.7	70.5±13.8	0.04	71.3±13.2	72.1±13.1	0.81
SBP supine	148.4±21.7	136.4±28.3	0.29	159.4±21.6	156.1±37.1	0.72
DBP supine	66.1±20.6	59.6±19.2	0.26	61.0±18.2	59.3±19.8	0.67
MAP supine	93.5±19.3	85.2±21.0	0.26	93.8±14.7	91.7±19.7	0.69
HR supine	77.9±13.7	69.1±13.9	0.02	69.4±12.1	69.2±12.2	0.91
SBP sit up	206.5±21.3	190.2±28.2	0.05	200.7±27.1	176.0±33.3	0.04
DBP sit up	98.4±24.5	90.7±24.9	0.04	85.1±27.1	77.7±28.2	0.26
MAP sit up	134.1±21.6	123.9±23.9	0.04	124.3±21.4	110.4±24.9	0.06
HR sit up	101.4±23.8	84.9±17.4	0.04	84.1±15.2	83.7±17.8	0.86
Valsalva						
Valsalva base MAP	120.2±19.7	107.6±18.6	0.07	113.0±22.4	102.4±30.2	0.26
Valsalva I MAP	146.8±22.0	126.6±22.0	0.03	131.5±18.6	116.5±27.3	0.05
Valsalva IIe MAP	110.6±17.6	93.3±17.0	0.03	94.5±16.6	91.5±24.3	0.63
Valsalva IIi MAP	131.6±25.4	109.8±30.8	0.04	112.4±26.0	115.1±28.5	0.82
Valsalva III MAP	107.0±26.4	89.3±28.2	0.12	89.4±27.0	81.5±26.6	0.38
Valsalva IV MAP	146.2±27.0	127.5±26.5	0.04	130.6±26.3	115.6±30.5	0.11

Significance is shown between time points. DBP indicates diastolic blood pressure; HR, heart rate; MAP, mean arterial pressure; SBP, systolic blood pressure.

With the Valsalva maneuver, the treatment group showed a significant reduction in MAP during phase I (11.5±17.4 mm Hg, *P*<0.05), phase IIe (15.4±19.3 mm Hg, *P*<0.05), phase IIi (19.1±25.2 mm Hg, *P*<0.05), and phase IV (17.8±24.1 mm Hg, *P*<0.05) at 6‐month follow‐up compared with baseline. In subjects who underwent the sham procedure, a significant reduction was only observed in MAP during phase I (10.6±29.6 mm Hg, *P*=0.05).

## Discussion

In this substudy of the WAVE IV trial, we demonstrate substantial and significant reduction in office BP compared with baseline in both treatment and sham groups, and reduction in ambulatory BP that was statistically significant in the sham group. Reasons for the failure of external ultrasound RDN to provide a significant reduction in BP over and above sham therapy have been previously described.^17^ It would be reasonable to propose that this novel technology actually did not achieve renal denervation based upon the BP response alone, but 2 lines of evidence support the hypothesis that renal nerve ablation did occur following active therapy in our study.

First, the decrease in HR in the RDN group both at rest and during postural maneuvers was significant, whereas it was not in the sham group. This is of interest because RDN has previously been shown to reduce HR.^19^ It is possible that HR changes are related to increased baroreflex sensitivity post‐RDN, and it has been previously demonstrated that impaired baroreflex sensitivity predicts response to catheter‐based RDN.^20^ In our study, however, there were no significant within‐group changes in baroreflex sensitivity or resting cardiac vagal tone from baseline compared with 6 months (Table S1). Together with the significant reduction in MAP during the sit up in the RDN group, this suggests that the observed HR changes following RDN might be attributable to a reduction in global sympathetic tone.

Second, the changes in autonomic function in the treatment group, and in particular reduction in MAP, noted during the Valsalva maneuver following RDN in our opinion suggests a successful renal nerve ablation procedure. The Valsalva maneuver is a commonly used physiological tool to assess autonomic function. The baroreceptor‐mediated tachycardia in phase II and bradycardia in phase IV are used as indices of cardiovagal integrity.^21^ Blood pressure recovery in late phase II and hypertensive response in phase IV are often used as indices of baroreceptor‐mediated sympathetic integrity.^22^

The sympathetic efferent nerves supply the capsulated organs in the splanchnic bed, namely, liver, spleen, and kidneys. Sympathetic stimulation plays an important role in α‐1‐adrenergic‐mediated contraction of these capsular organs. This drives splanchnic autotransfusion—equivalent to a blood volume reservoir—in the splanchnic vascular bed, which in turn increases sympathetic‐driven venous return to the heart whenever it is below a regulated level.^16^

The mechanism of BP changes during the Valsalva maneuver is described elsewhere.^23^ There is evidence of volume receptors and/or baroreceptor modulation of the volumes of encapsulated organs in the splanchnic vascular bed through sympathetic‐mediated constriction during reduced venous return.^24^ There is also evidence of left atrial volume receptors stimulated by atrial dilation that modulates renal sympathetic activity in animal studies.^25^ Since there is evidence of plasma volume–related BP responses to the Valsalva maneuver in humans, the regulatory mechanism is the same as observed in animal studies.^26^ There are in addition studies of BP changes in humans during the Valsalva maneuver after pharmacological blockade of α‐1‐ and β‐1‐adrenergic receptors and other functions of the autonomic nervous system.^23^ It is therefore clear that the late phase II, marked by the recovery of BP during active positive intrathoracic pressure, is caused by volume receptor–mediated increase in sympathetic outflow leading to α‐1‐adrenergic‐mediated vasoconstriction resulting in splanchnic autotransfusion as a consequence of the contraction of capsulated organs such as the liver, spleen, and kidneys. This is accompanied by cardiac sympathetic stimulation and tachycardia.

In this study, we have shown attenuation of this α‐1‐adrenergic‐mediated BP recovery in late phase II and phase IV of the Valsalva maneuver in the RDN group that is not seen in the sham group and has never been previously demonstrated. This shows reduction in greater splanchnic nerve sympathetic activity, because there is no change in total blood volume or BP. Of note, in animal models, total SNS tone in the body is directly correlated with MAP within a certain BP range.^27^ In this cohort of subjects, the baseline MAP in the 2 subgroups was very similar. There was no significant change in resting MAP in the RDN group or sham group postprocedure, suggesting no change in the total SNS activity with RDN.

Previous studies using RDN to lower BP have quantified change in SNS activity in the body with changes in muscle sympathetic nerve activity and norepinephrine spillover, but even these techniques have their limitations and are not ideal measures for assessing procedural success.^28^ Measuring peripheral sympathetic drive by microneurography is invasive and technically demanding for both operator and the patient. In addition, the information provided is limited to regional sympathetic neural activity. Given the heterogeneity of system‐specific sympathetic innervations, muscle sympathetic nerve activity may not necessarily reflect changes in global sympathetic tone. Muscle sympathetic nerve activity measurements can be affected by observer variability by 9% and have inherent bias against low levels of sympathetic activity.^28^ Norepinephrine spillover is an indirect index of sympathetic neural activity and is influenced by regional differences in sympathetic outflow to different vascular beds. Also, different environmental stress triggers lead to variable regional sympathetic outflow and hence, influences the levels of norepinephrine spillover. Newer methods of assessing sympathetic innervation in the context of RDN include the ConfidenHT mapping system (http://ClinicalTrials.gov Identifier: NCT02777216) and direct neural monitoring from Autonomix Medical (http://www.autonomixmed.com), which are currently under evaluation and remain to be proven with no published data to date.

Although the WAVE IV study was terminated early, it is important to note that a sizeable sham effect in device therapy of hypertension studies is well recognized and clearly this needs to be explored further.^2^, ^29^ It may be considered that this finding acts as a warning that interventional therapies have not failed as antihypertensive strategies if they demonstrate significant absolute ambulatory BP reduction (>6 mm Hg) compared with baseline, but they may not necessarily succeed against sham.^30^ Sham effects can persist up to 6 months postprocedure as seen in the WAVE IV and other studies. In our substudy, we demonstrate that the commonly used BP‐lowering end point is just 1 of several cardiovascular outcomes of RDN, as we have demonstrated effective renal nerve ablation following RDN with changes in both HR and in the Valsalva maneuver that were not seen with sham.

Our findings should be tested a priori in a larger randomized controlled study before making strong recommendations about how to use these markers to document success, and may furthermore lead to improved patient selection for RDN therapies because those subjects who demonstrate exaggerated restoration of MAP (>15 mm Hg above baseline) in phase 2i of Valsalva, indicative of raised baseline splanchnic sympathetic tone, are likely to exhibit an increased response to renal nerve ablation. Given that our method is noninvasive, this could be thereafter adopted at scale in addition to its use as a marker of procedural success.

Renal denervation has moved some way forward since it was first demonstrated to be effective in open‐label studies almost a decade ago. Since then there have been considerable advances in our understanding of renal neural microanatomy and how best to achieve effective endovascular renal SNS ablation. Now that a recent proof of concept study has demonstrated convincingly that RDN is effective at lowering BP in moderate hypertension compared with sham, it has become more important than ever before to demonstrate procedural success.^31^ Our approach using the Valsalva maneuver is relatively straightforward and reproducible and illustrates convincingly that RDN can attenuate BP responses to the Valsalva maneuver, which was not the case with a sham procedure. Just as importantly, we show that despite having no effect on renal SNS innervation, a sham procedure has profound effects on office and ambulatory BP, which has significant implications for clinical trial designs of the future.

### Limitations

Our study has its limitations. It was undertaken as a substudy of the WAVE IV trial but the study assessments (including autonomic function tests) and the analysis was done in a blinded fashion to exclude bias. Our sample size was small and the study was not powered to assess changes in autonomic function tests between the groups. Although pre‐existing medications might have influenced the results (particularly in the case of drugs interfering with SNS activity such as diuretics and β‐blockers), they were evenly matched between the 2 groups and it would have been unethical to alter antihypertensive medications in order to do autonomic assessments. Importantly, in the WAVE IV study, overall adherence with medications was demonstrated to be ≈80% for both groups at baseline and did not change significantly at the 6‐month follow‐up visit, suggesting that medication changes were not responsible for the changes in BP, HR, or Valsalva parameters in either group.

## Author Contributions

Lobo was the principal investigator of this trial. Saxena and Shour wrote this report and all other authors contributed to data collection and/or revisions to the manuscript.

## Sources of Funding

The WAVE IV study was funded by Kona Medical. Autonomic testing (equipment and personnel) were funded by Barts Charity. M. Saxena, MDL, DJC, VK, AG, AZ, AB and JP are supported in part by Barts NIHR BRC and this work forms part of the research portfolio for the National Institute for Health Research Barts Biomedical Research Centre.

## Disclosures

Dr Lobo has received a modest research grant from Medtronic, modest honoraria from Cardiosonic, CVRx, Kona Medical and St. Jude Medical, and modest fees for consulting and advisory board participation from ROX Medical. Professor Dr med. Schmieder has received a modest research grant and modest speaker bureau fees from Kona Medical, Medtronic, and Recor. The remaining authors have no disclosures to report.

## Supporting information


**Data S1.** Supplemental Methods.
**Table S1.** Within‐Group Changes in Resting Cardiac Vagal Tone (CVT) and Baroreflex Sensitivity (BRS) Between Baseline and 6 Months Postprocedure
