# Supplementary material for: Attenuation of Splanchnic Autotransfusion Following Noninvasive Ultrasound Renal Denervation: A Novel Marker of Procedural Success
Source: J Am Heart Assoc. 2018 Jun 12;7(12):e009151. doi: 10.1161/JAHA.118.009151 (PMC6220552; doi:10.1161/JAHA.118.009151)
Supplement: Supplementary file 1 — Data S1. Supplemental Methods. Table S1. Within‐Group Changes in Resting Cardiac Vagal Tone (CVT) and Baroreflex Sensitivity (BRS) Between Baseline and 6 Months Postprocedure [file JAH3-7-e009151-s001.pdf]

SUPPLEMENTAL MATERIAL

Data S1.

Supplemental Methods

The WAVE IV Study was carried out at 13 institutions across 5 countries (Czech Republic, Germany, New Zealand, Poland, and the UK). All patients included in the study provided written informed consent. The trial received ethical approval from the National Research ethics Committee and was conducted in accordance with the Declaration of Helsinki and its amendments. The study was registered at www.clinicaltrials.gov; NCT02029885. The trial was halted after an interim analysis on July 19, 2016. This was as a result of a lack of evidence of any antihypertensive efficacy of the externally delivered focused ultrasound in the RDN group over the sham control. It appeared to be futile to continue and of note, there was no safety concern to stop the trial.

Screening and baseline period

At the initial screening visit, eligibility was assessed by taking a full medical history, including current and past antihypertensive medication regimens. At this first baseline visit BP measurements were taken to determine if the mean office SBP was ≥160 mmHg. Patients were provided with a study-specific medication diary in which to record their compliance with their antihypertensive medications.

Four weeks later, patients attended the second baseline visit, where further BP measurements were taken to assess if the mean SBP remained at ≥160 mmHg. Medication adherence during the previous 4 weeks was evaluated using pill counting and by reviewing the diary. Pill counts were required to have been within 80% of expected levels for

continued inclusion in the study. ABPM was performed in each patient with a certified device to determine if the mean 24 h SBP was ≥135 mmHg, as per the inclusion criteria.

Two weeks thereafter, the patient attended the third baseline visit. Medication adherence was further evaluated and routine laboratory tests were performed. A urine sample was collected for assessment of medication adherence but this additional measure was introduced in the protocol after the study was already running. Kidney function was evaluated by measurement of the estimated glomerular filtration rate (eGFR). Duplex renal ultrasound and CT angiography were performed to visualize renal artery flow velocity and to rule out renovascular causes of hypertension. BP measurements were performed to determine if the mean SBP was ≥160 mmHg. Furthermore, if there was a difference of ≥15 mmHg between the second and third visit, the patient was required to attend a further visit 2 weeks later. At this point, if the SBP was still ≥15 mmHg away from the value recorded at least the second visit, the patient was excluded from the study due to instability of baseline BP. Of note, change in office BP was the primary objective following the Symplicity HTN-3 study design, the results of which had not been published at the time when first patient were included.[1]

Once all inclusion and exclusion criteria were confirmed, patients were centrally randomized to the intervention. Before patients were sent for intervention, all eligible patients at our centre had a detailed autonomic function test as standard of care in patients assessed for device based therapies of hypertension.

Intervention

Patients were centrally randomized in a 1:1 ratio to receive either the active RDN treatment or the sham control, both administered using the Surround Sound System. The treatment consisted of bilateral RDN using therapeutic levels of ultrasound energy. The sham consisted of bilateral sham treatment using diagnostic levels of ultrasound energy. Group assignment was carried out by the delivery of an encrypted code directly to the Surround Sound System, which then applied the respective amount of energy to the patient. Both subjects and investigators therefore remained blinded to the randomisation. In order to mask variations in pain response, all patients were given conscious sedation regardless of randomisation assignment. Blinding was assessed by separately questioning the investigator and the patient to determine if they thought they had received the active treatment or the sham.

The Kona Surround Sound system comprises a generator, a water conditioner, and a treatment module with an imaging probe, all contained in a single mobile patient platform. The imaging probe is a diagnostic ultrasound array that is used to locate the renal artery and associated structures. Real-time motion tracking allows for accuracy in the location of the applied energy. The treatment module additionally contains a phased array therapeutic ultrasound transducer which delivers the therapy.

Measurement of Cardiac Vagal Tone and Baroreflex Gain

The non-invasive NeuroScope™ method was used (MediFit Instruments Ltd, London), to evaluate autonomic neurophysiology, as has been previously described in the monitoring of brainstem autonomic functions in routine clinical examination of neurodevelopmental disorders such as Rett syndrome and Autistic Spectrum Disorders [2-5].

A non-invasive continuous index of cardiac vagal tone (CVT) defined as, "pulse synchronized phase shifts in consecutive cardiac cycles" is a form of pulse interval jitter was quantified in real time by the NeuroScope as previously described [6]. CVT is also continuously measured in real time and allows measurement of sudden or rapid changes in response and can be used to monitor the activity of the brainstem parasympathetic system during rapid changes in cardiovascular control. The CVT is measured and quantified in clinically validated units of a linear vagal scale (LVS) where the zero reference point of the scale is equivalent to full atropinisation in human subjects [7].

Baroreflex sensitivity (BRS), measured in ms/mmHg, was recorded as a measure of central cardiovascular regulation, by the NeuroScope as previously described [8]. This index is defined as the increase in pulse interval per unit increase in systolic blood pressure and quantifies the negative feedback control of blood pressure beat by beat. The method allows detection of rapid changes in baroreflex gain in real time within a continuous measurement. It therefore facilitates the crucial measurements of sudden changes in autonomic regulatory responses and is also useful for monitoring whether or not this

sympathoregulatory function of the brainstem is fully engaged during cardiovascular control at any given moment. Baroreceptor control of the heart has been modelled as a closed loop feedback system with a delay line in the sympathetic nervous system that makes it resonate at 0.1 Hz [9]. Hence the effect of one perturbation is diffused over a period of at least 10 s, which is twelve cardiac cycles if the HR is 72 beats min^{-1}. For normal heart rates (between 60-100 beats per minute), it is reasonable to use an average of 20 beats to cover a single perturbation.

The baseline autonomic status (CVT and BRS) was recorded in the supine position for at least three minutes with the mean BP varying by less than 5 mmHg. The conditions for autonomic function testing are described in the main manuscript.

Statistics

When looking at within group differences between time points, two-tailed dependent student's t tests were used. A significant difference was defined as having a p value < 0.05.

Table S1. **Within group changes in resting cardiac vagal tone (CVT) and baroreflex sensitivity (BRS) between baseline and 6 months post procedure.**

	Treatment			Sham		
	Baseline	6 Months	*P* value	Baseline	6 Months	*P* value
Resting CVT	4.07 ± 3.41	5.71 ± 5.33	0.14	5.81 ± 3.01	4.79 ± 1.58	0.19
BRS (supine)	3.64 ± 2.87	4.16 ± 3.01	0.31	3.08 ± 1.91	2.95 ± 1.83	0.71

* Values are shown as mean ± SD

† Significance is shown between time points

‡ CVT = cardiac vagal tone

§ BRS = baroreflex sensitivity

Supplemental References:

1. Bhatt DL, Kandzari DE, O'Neill WW, D'Agostino R, Flack JM, Katzen BT, Leon MB, Liu M, Mauri L, Negoita M, Cohen SA, Oparil S, Rocha-Singh K, Townsend RR, Bakris GL, Investigators SH-. A controlled trial of renal denervation for resistant hypertension. *The New England journal of medicine*. 2014;370:1393-1401.

2. Julu PO, Kerr AM, Apartopoulos F, Al-Rawas S, Engerstrom IW, Engerstrom L, Jamal GA, Hansen S. Characterisation of breathing and associated central autonomic dysfunction in the rett disorder. *Arch Dis Child*. 2001;85:29-37.

3. Ming X, Patel R, Kang V, Chokroverty S, Julu PO. Respiratory and autonomic dysfunction in children with autism spectrum disorders. *Brain Dev*. 2016;38:225-232.

4. Julu PO, McCarron MO, Hansen S, Barnes A, Jamal GA, Ballantyne JP. Selective defect of baroreflex blood pressure buffering with intact cardioinhibition in a woman with familial aniridia. *Neurology*. 1997;49:1705-1708.

5. Julu PO, McCarron MO, Hansen S, Job H, Jamal GA, Ballantyne JP. Complex regional pain syndrome with selective emotional sudomotor failure. *Eur J Neurol*. 2000;7:351-354.

6. Little CJ, Julu PO, Hansen S, Reid SW. Real-time measurement of cardiac vagal tone in conscious dogs. *The American journal of physiology*. 1999;276:H758-765.

7. Puri BK, Shah M, Monro JA, Kingston MC, Julu PO. Respiratory modulation of cardiac vagal tone in lyme disease. *World J Cardiol*. 2014;6:502-506.

8. Julu PO, Cooper VL, Hansen S, Hainsworth R. Cardiovascular regulation in the period preceding vasovagal syncope in conscious humans. *The Journal of physiology*. 2003;549:299-311.

9. deBoer RW, Karemaker JM, Strackee J. Hemodynamic fluctuations and baroreflex sensitivity in humans: A beat-to-beat model. *The American journal of physiology*. 1987;253:H680-689.
